# Heme oxygenase-1 repeat polymorphism in septic acute kidney injury

**DOI:** 10.1371/journal.pone.0217291

**Published:** 2019-05-23

**Authors:** Laura M. Vilander, Suvi T. Vaara, Kati M. Donner, Päivi Lakkisto, Mari A. Kaunisto, Ville Pettilä, the FINNAKI Study Group

**Affiliations:** 1 Division of Intensive Care Medicine, Department of Anesthesiology, Intensive Care and Pain Medicine, University of Helsinki and Helsinki University Hospital, Helsinki, Finland; 2 Institute for Molecular Medicine Finland (FIMM), University of Helsinki, Helsinki, Finland; 3 Department of Clinical Chemistry, University of Helsinki and Helsinki University Hospital, Helsinki, Finland; 4 Minerva Foundation Institute for Medical Research, Helsinki, Finland; National Cancer Institute, UNITED STATES

## Abstract

Acute kidney injury (AKI) is a syndrome that frequently affects the critically ill. Recently, an increased number of dinucleotide repeats in the *HMOX1* gene were reported to associate with development of AKI in cardiac surgery. We aimed to test the replicability of this finding in a Finnish cohort of critically ill septic patients. This multicenter study was part of the national FINNAKI study. We genotyped 300 patients with severe AKI (KDIGO 2 or 3) and 353 controls without AKI (KDIGO 0) for the guanine–thymine (GTn) repeat in the promoter region of the *HMOX1* gene. The allele calling was based on the number of repeats, the cut off being 27 repeats in the S–L (short to long) classification, and 27 and 34 repeats for the S–M–L_2_ (short to medium to very long) classification. The plasma concentrations of heme oxygenase-1 (HO-1) enzyme were measured on admission. The allele distribution in our patients was similar to that published previously, with peaks at 23 and 30 repeats. The S-allele increases AKI risk. An adjusted OR was 1.30 for each S-allele in an additive genetic model (95% CI 1.01–1.66; *p* = 0.041). Alleles with a repeat number greater than 34 were significantly associated with lower HO-1 concentration (*p*<0.001). In septic patients, we report an association between a short repeat in *HMOX1* and AKI risk.

## Introduction

Acute kidney injury (AKI) is a multifactorial syndrome that frequently accompanies critical illness. In a Finnish intensive-care-unit (ICU) cohort, the incidence of AKI was 39% [1]. Clinical risk factors alone fail to reliably predict the development and severity of AKI. Therefore, common genetic variants, genetic polymorphisms, have been studied in association with development and outcome of AKI, but no conclusive evidence about the role of polymorphisms exists.

Disturbances in iron metabolism are associated with inflammation and oxidative stress and have been suggested to participate in the pathogenesis of AKI [2–4]. A recent study presented an association between a repeat polymorphism in the heme oxygenase-1 (*HMOX1*) gene and the development of AKI in adults undergoing cardiac surgery [5]. This dinucleotide (GTn, guanine–thymine) repeat polymorphism in the promoter region of *HMOX1* has been shown to influence heme oxygenase (HO-1) levels [6,7]. Saukkonen and colleagues [6] have deciphered the reference range for HO–1 plasma concentration (0.66–2.39ng/mL) in 58 healthy subjects. Moreover, they presented that the plasma concentrations of this ubiquitously expressed enzyme are elevated in the critically ill [6].

In this study, we aimed to investigate whether the previously reported association between *HMOX1* repeat polymorphism and AKI development [5] can also be seen in our study population of critically ill septic patients. Additionally, we sought to verify the impact of the polymorphism on their protein levels.

## Materials and methods

### Study population

This study was part of a national, multicenter, prospective, observational FINNAKI study conducted in 17 Finnish ICUs in the years 2011–2012. The study population consisted of adult Finns (>18 years). We enrolled all patients admitted due to emergency admission of any expected length, as well as elective postoperative patients with an expected stay of >24 hours. The current analysis included only patients with sepsis. Genetic samples were collected on admission. A written informed consent was obtained from the patient or the next of kin as soon as possible, however the initiation of the study for each participant was not delayed due to the deferred consent procedure. In this procedure the participant was enrolled to the study were there no exclusion criteria and the inclusion criteria were fulfilled on admission to the ICU. Should the participant or the next of kin decline the study, the data gathered thus far were removed. Exclusion criteria are listed in the supporting information (S1 Appendix). The study was approved by the Ethics Committee of the Department of Surgery in Helsinki University Hospital and adheres to the Declaration of Helsinki.

### Definitions

AKI was defined according to Kidney Disease: Improving Global Outcomes (KDIGO) criteria [8]. To strengthen our findings, we defined the AKI phenotype as KDIGO stages 2 or 3, and we chose to exclude patients with stage-1 AKI from the analysis [9]. We defined sepsis according to American College of Chest Physicians/Society of Critical Care Medicine (ACCP/SCCM) criteria [10].

### Data collection

Database maintained by Finnish Intensive Care Consortium (FICC) collected and provided routine data (Tieto Ltd, Helsinki, Finland). Moreover, on admission as well as daily for 5 days we collected study-specific data in a standardized case report form (CRF). We measured plasma creatinine daily and urine output hourly. An automated calculator provided the AKI stage calculations continuously.

### Genotype analysis

Blood samples for DNA extraction were collected on admission and after separation of plasma, stored at –80°C. DNA was isolated using a Chemagic 360 instrument (Perkin Elmer, Baesweiler, Germany), based on magnetic bead technology. A Chemagic DNA Blood10k Kit was used according to manufacturer's instructions. DNA samples were diluted to 12ng/μl concentration for genotyping.

The GTn repeat sequence in the *HMOX1* gene promoter was first amplified by polymerase chain reaction (PCR) using a 5’FAM–labeled forward primer 5’–FAM–AGAGCCTGCAGCTTCTCAGA–3’ and a reverse primer 5’–ACAAAGTCTGGCCATAGGAC–3’. The sizes of the PCR products were determined with fragment analysis using an ABI3730xl DNA Analyzer (Applied Biosciences, Thermo Fisher Scientific, Vernon Hills, IL). Allele calling was based on the GeneMapper Software version 4 (Applied Biosciences, Thermo Fisher Scientific, Vernon Hills, IL) and visual inspection of the data by two investigators (LV and MK). As quality control, 2 duplicate samples, and 2 water controls were included on each plate. Six (0.7%) samples were discarded because of poor quality. As a further quality control step, the GT repeat numbers were verified by capillary sequencing of 15 homozygote samples.

The alleles were classified into genotype groups in two different ways, based on the frequency distribution of the alleles, as previously described [5]. In the two–class classification, 27 repeats was used as the cutoff [5,11]: <27 repeats were designated short (S), whereas alleles with ≥27 repeats were designated long (L). Accordingly, three distinct genotypes, SS, SL, and LL, were used in the analyses. The minor allele (S) frequency was 0.35. We performed an additional analysis using three length classes [6,12] for repeat classification (S < 27 ≥ M < 34 ≤ L_2_, as separation for L), along with genotypes SS, SM, MM, SL_2_, ML_2,_ and L_2_L_2_.

### HO–1 plasma concentrations

From ethylene diamine tetra acetate (EDTA) anticoagulated plasma collected on admission, HO-1 enzyme concentration was measured using an enzyme–linked immunosorbent assay (ELISA). The detailed description of the method and its quality control are provided in the supporting information (S2 Appendix).

### Power calculation

The original study demonstrating an association between *HMOX1* GTn polymorphism and cardiac surgery related AKI [5] reported odds ratios (OR) of AKI for L–allele 1.25 and for LL genotype 1.58. Based on this study, we estimated that approximately 600 patients (300 without AKI) would be an adequate sample size to provide an 80% power to detect an association with a *p*-value significance level of 0.05. Retrospectively, with these odds ratio assumptions and the sample size, allele frequency, as well as AKI incidence from our data, with a *p-*value of 0.05, the power of our study setting with allelic test was 0.89 and with genotype test 0.83 (by Genetic Power Calculator [13]).

### Statistical methods

Statistical analyses for the demographic data and for the comparisons between genotype groups were performed with SPSS Statistics version 22 (IBM Corp., Armonk, NY, USA). We present OR (odds ratios) with 95% CIs (confidence intervals), using Fishers exact test, for categorical variables and Kruskal–Wallis test for continuous variables. We present Cochran–Armitage test for trend for ordinal genotype trend test. We tested for Nagelkerke pseudo R^2^ for the logistic regression model with covariates, and with the model including the genotype (see supporting information (S3 Appendix)). Logistic regression with additive, recessive and dominant genetic models were performed with PLINK software [14]. We considered *p*-values <0.05 significant.

## Results

**Fig 1:**
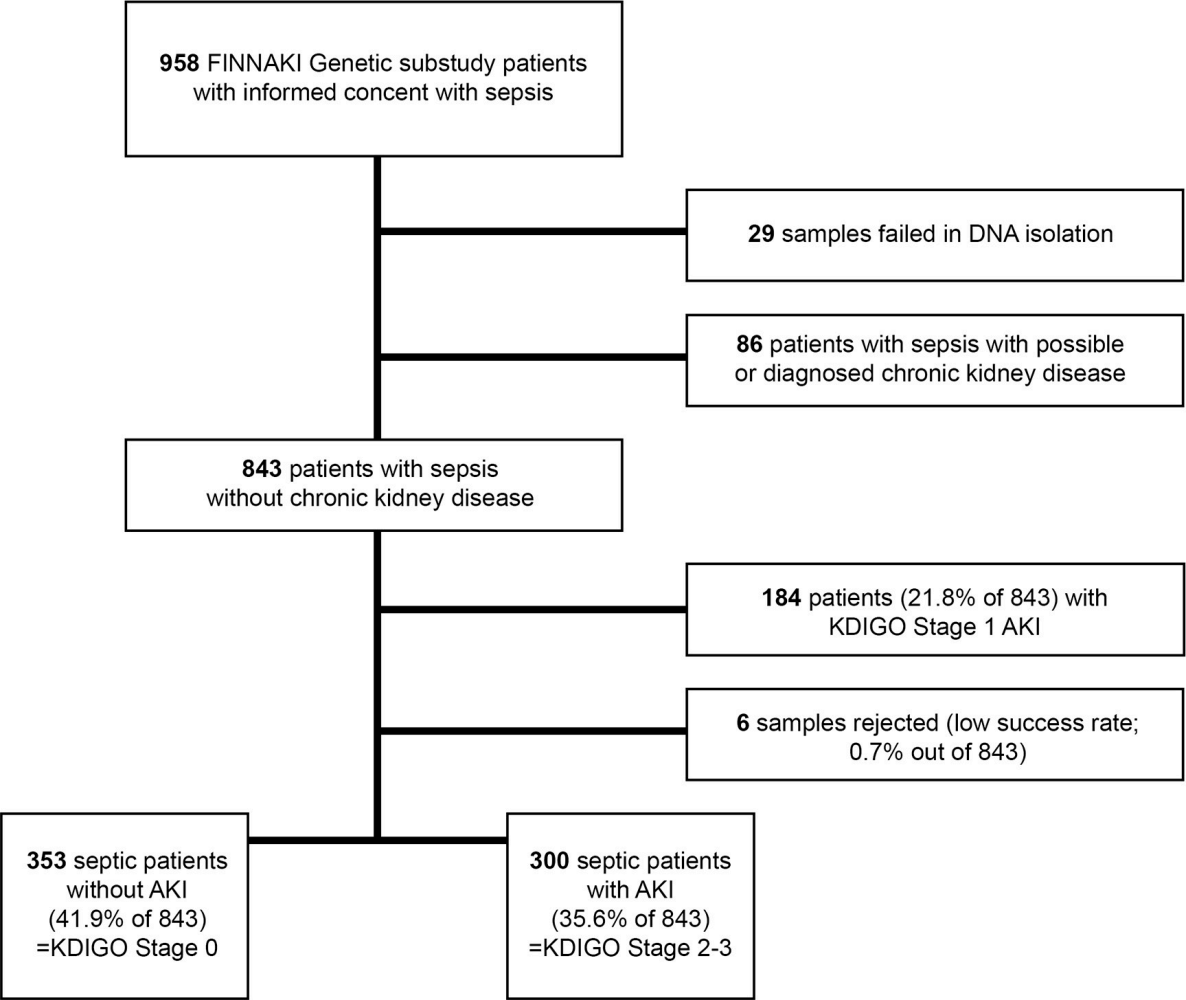
Study flow chart. Abbreviations: FINNAKI, Finnish acute kidney injury; DNA, deoxyribonucleic acid; KDIGO, Kidney Disease: Improving Global Outcomes; AKI, acute kidney injury.

Altogether 653 patients with sepsis were successfully genotyped for *HMOX1*-promoter polymorphism. Their demographic and clinical data are presented in Table 1. Of these patients, 300 had KDIGO stage 2 or 3 AKI and 353 did not have AKI (KDIGO stage 0) (Fig 1). Patients with KDIGO stage 1 (N = 189) AKI were not included in the analyses.

**Table 1:** Demographic data.

	Data available	All patients (N = 653)	No AKI (n = 353)	AKI (n = 300)	*p*-value
**Age (Y)**	653	63 (53–74)	63 (51–72)	64 (54–75)	0.055
**Gender (male)**	653	418 (64)	234 (66)	184 (61)	0.192
**BMI (kg/m2)**	651	26.5 (23.5–29.7)	26.0 (23.1–29.2)	27.3 (24.4–30.8)	0.001
**Co–morbidities**					
**Arterial hypertension**	651	323 (50)	163 (46)	160 (53)	0.084
**Diabetes**	653	150 (23)	68 (19)	82 (27)	0.015
**Arteriosclerosis**	648	76 (12)	35 (10)	41 (14)	0.178
**COPD**	649	64 (10)	45 (13)	19 (6)	0.008
**Chronic liver disease**	647	42 (7)	18 (5)	24 (8)	0.151
**Systolic heart failure**	649	64 (10)	40 (11)	24 (8)	0.186
**Thromboembolism**	649	42 (7)	25 (7)	17 6)	0.524
**Rheumatic disease**	648	43 (7)	25 (7)	18 (6)	0.636
**Baseline plasma creatinine (μmol/l)**	653	79.0 (67.0–93.3)	80.0 (68.0–94.0)	77.2 (66.5–93.0)	0.341
**Admission**					
**Emergency**	648	635 (98)	342 (98)	293 (98)	1.000
**Operative**	652	154 (24)	87 (25)	67 (22)	0.518
**Cardiac surgery**	653	12 (2)	8 (2)	4 (1)	0.561
**SAPS II score 24h without renal and age points**	649	25 (18–33)	24 (17–30)	26 (20–37)	<0.0001
**White blood cells, maximum (10^9^/l)**	584	11.9 (7.7–12.7)	11.7 (7.9–16.4)	12.2 (7.5–17.8)	0.450
**Platelets, minimum (10^9^/l)**	632	189.5 (128.0–265.0)	196.0 (141.0–268.0)	184.0 (111.0–261.0)	0.040
**Plasma bilirubin, maximum (μmol/l)**	305	14.0 (7.0–25.5)	13.0 (7.0–22.0)	14.0 (8.0–32.0)	0.150

Continuous variables distribute non–normally and are reported as median (interquartile range) and categorical variables as number (percentage). Abbreviations: AKI, acute kidney injury; BMI, body mass index; COPD, chronic obstructive pulmonary disease; SAPS, simplified acute physiology score.

**Fig 2:**
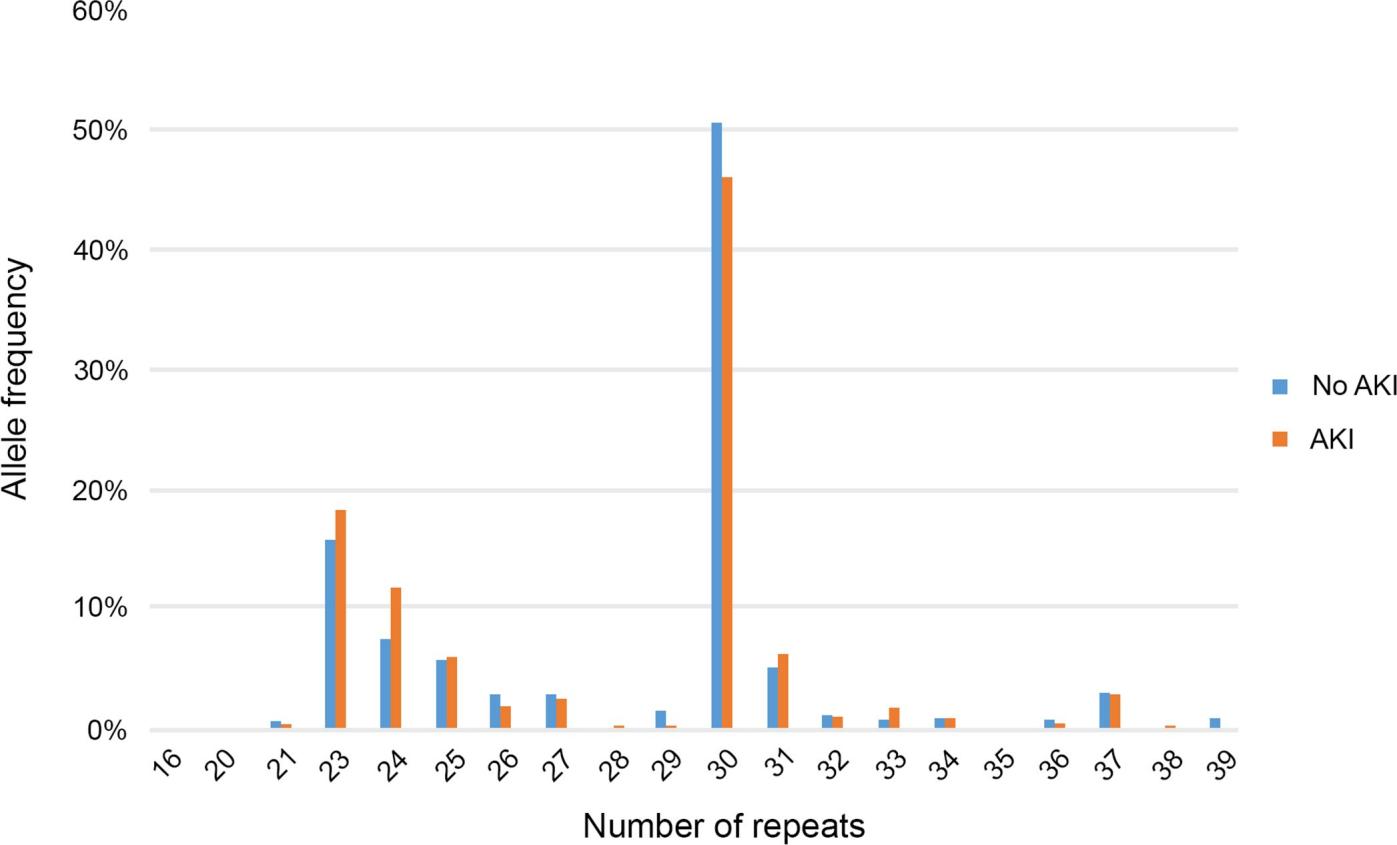
Distribution of alleles with (GT)_n_ repeats in patients with and without acute kidney injury (AKI). The number of repeats ranged from 16 to 39.

The allele distribution of the GT repeats is shown in Fig 2 and is similar to previously reported values. The most common alleles had 23 repeats (16.8%) and 30 repeats (48.5%).

### Descriptive statistics

**Fig 3:**
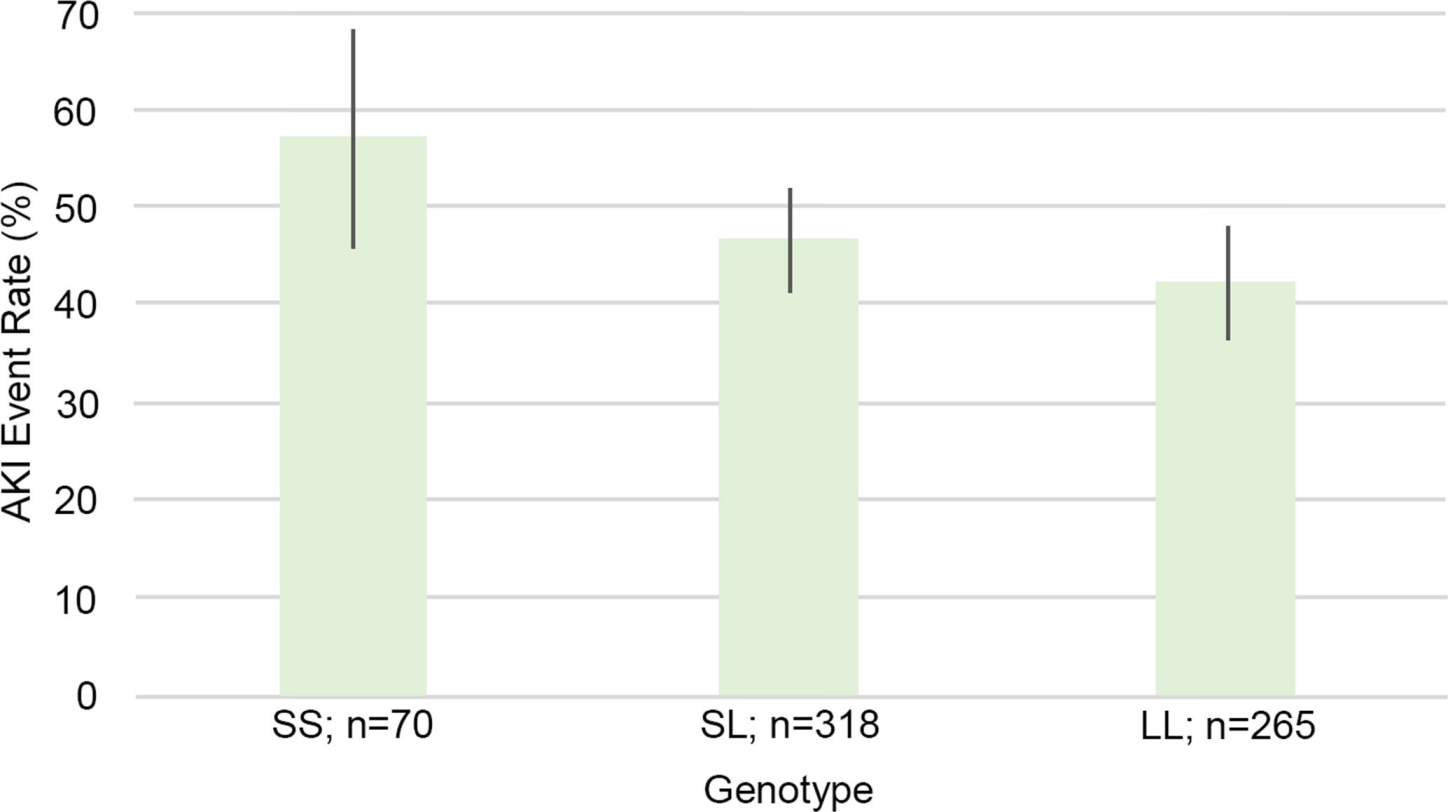
Acute kidney injury (AKI) event rate according to genotype (SS, SL, or LL).

**Fig 4:**
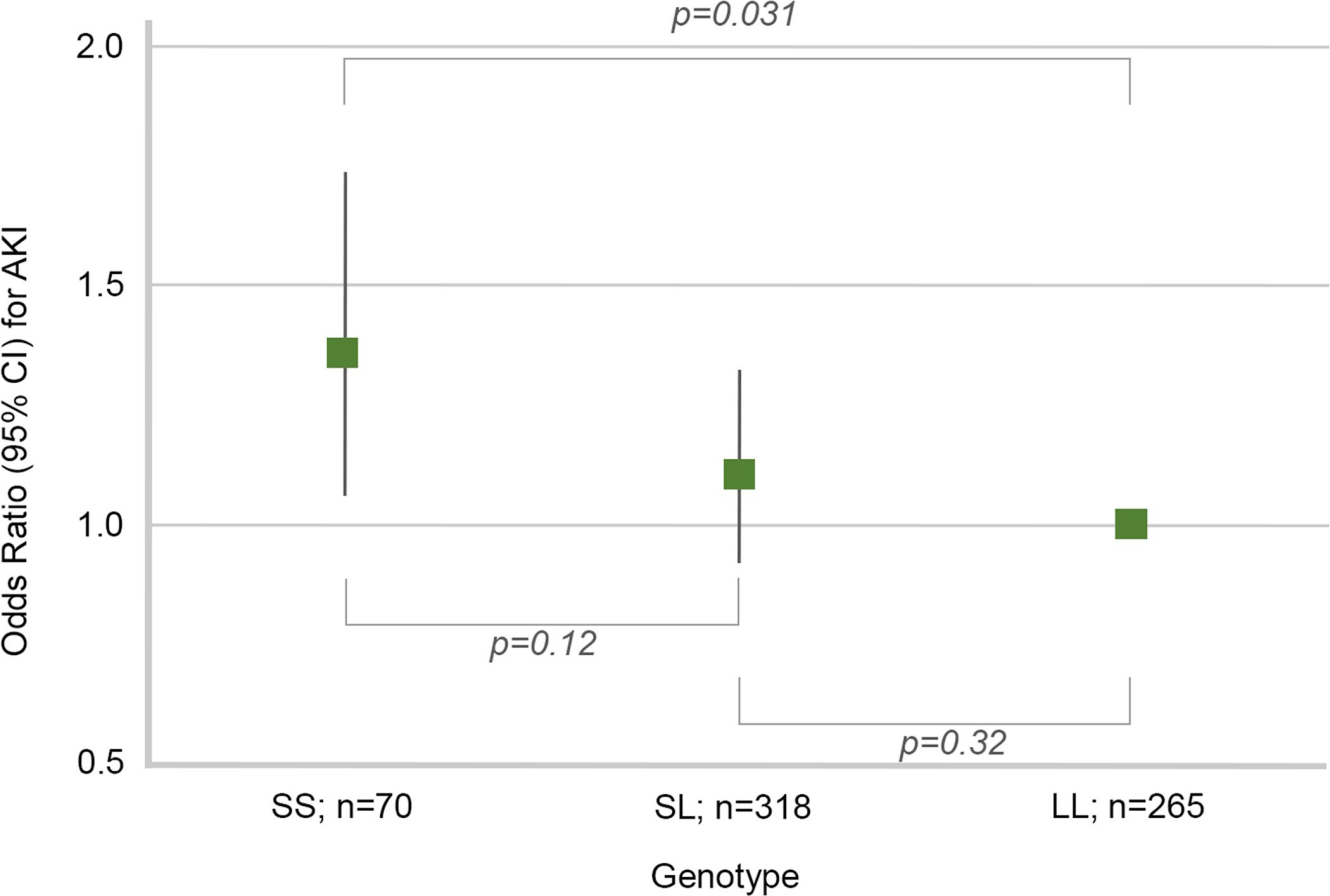
Odds ratio (OR) of acute kidney injury (AKI) according to genotype (SS, SL, or LL).

There were 70 patients with an SS genotype, 318 patients with an SL genotype, and 265 patients with an LL genotype. Of these patients, 40 (57%) within SS, 148 (47%) within SL, and 112 (42%) within LL had KDIGO stage 2 or 3 AKI (Fig 3) (*p* for trend 0.034, Cochran Armitage test for trend). When only the SS and LL genotypes were compared, the SS genotype had an OR of 1.35 of AKI (95% CI 1.06–1.73, *p* = 0.031) (Fig 4).

**Fig 5:**
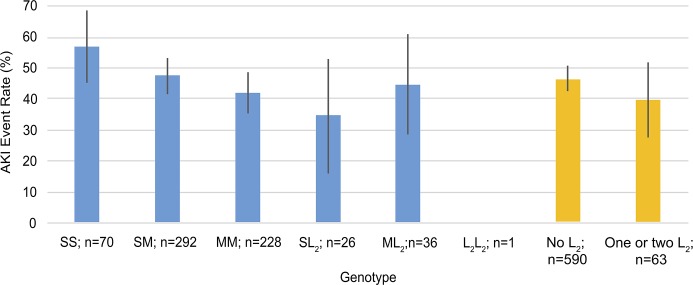
Acute kidney injury (AKI) event rate according to genotype; three-class classification. Allele lengths are: S <27 repeats, 27≤ M <34, L_2_ ≥34. The blue columns each illustrate the event rate within a genotype, whereas the orange columns illustrate the event rate in all the genotypes without a very long allele in comparison to genotypes with one or more very long alleles.

The event rates by genotype according to the three–class repeat classification are presented in Fig 5 (*p* for trend 0.029, Cochran Armitage test for trend). When genotypes with either one or two long L_2_ alleles (≥ 34 repeats) were compared to all the other genotypes We found no significant difference in incidence of AKI between these groups (NS, Fig 5).

The Nagelkerke R Square statistic for covariates alone was 0.126. When the two-class or three-class classification genotype was added, the Nagelkerke value was 0.134 and 0.138, respectively. The genotype groups did not differ in disease severity according to SAPS II score without age and renal points (see supporting information (S4 Appendix) for detailed results).

### Logistic regression

The S-allele was a minor allele with a frequency of 0.35 in the AKI patients, and the risk allele for AKI. In an additive genetic model, each S-allele increased the risk of AKI with an OR of 1.29 (95% CI 1.02–1.64, *p* = 0.034). Adjustment for age, gender, body mass index (BMI), diabetes, hypertension, chronic obstructive pulmonary disease (COPD), simplified acute physiology score II (SAPS II) without points from age and kidney function, and platelet count, in multivariate logistic regression with additive genetic model did not change the direction or the magnitude (OR 1.30, 95% CI 1.01–1.66; *p* = 0.041). The results for different genetic models are presented in Table 2.

**Table 2:** Odds ratios for HMOX1 risk alleles and acute kidney injury in different genetic risk models.

AKI risk		Unadjusted		Adjusted	
genetic model	risk allele	odds ratio (95% CI)	*p*-value	odds ratio (95% CI)	*p*-value
**additive**	S	1.292 (1.019–1.639)	0.034	1.296 (1.011–1.662)	0.041
**recessive**	S	1.656 (1.004–2.733)	0.048	1.626 (0.962–2.746)	0.069
**dominant**	S	1.284 (0.937–1.759)	0.119	1.301 (0.936–1.807)	0.117

Abbreviations: AKI, acute kidney injury; CI, confidence interval.

### HO-1 plasma concentration analysis

**Fig 6:**
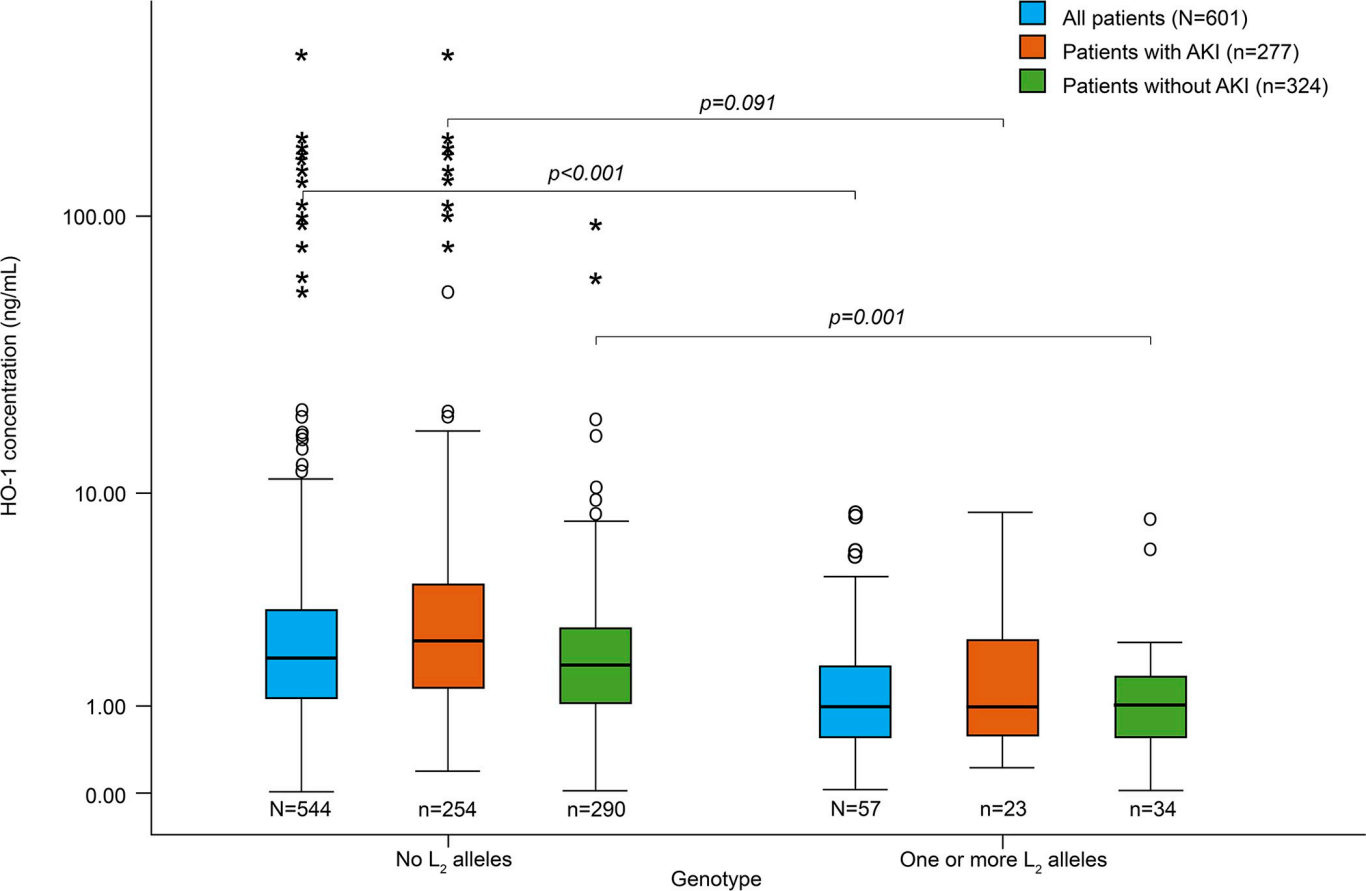
Heme oxygenase-1 (HO-1) concentration according to presence of L_2_-allele (≥34 repeats) and acute kidney injury (AKI).

Of the 653 patients, 601 (92%) were successfully sampled for HO-1 plasma concentration. HO-1 was significantly higher in patients with AKI (2.2ng/mL vs 1.7ng/mL, *p* = 0.001). The median plasma concentration of HO-1 did not differ according to *HMOX1* repeat polymorphism genotype when the two-class repeat classification was used (*p* = 0.35, see supporting information (S5 Appendix) for more detailed results). However, according to the three-class repeat classification, the median HO-1 concentration differed between the genotypes (*p* <0.001). The patients with at least one L_2_-allele had significantly lower median HO-1 concentration when compared to the other genotypes with no L_2_ alleles (1.0ng/mL vs 2.0ng/mL, *p*<0.001). In addition, this finding is significant when only the patients without AKI (1.0ng/mL vs 1.8ng/mL, *p*<0.001), and the slightly smaller (n = 277) group of patients with AKI (1.0ng/mL vs 2.4ng/mL, *p* = 0.004) were analyzed separately (Fig 6).

## Discussion

In critically ill septic patients we report that the S-allele (<27 repeats) in the *HMOX1* promoter region is associated with development of AKI. This finding is in contrast with that previously reported for cardiac-surgery related AKI [5]. The longest repeats (>34) were significantly associated with lower HO-1 concentrations.

Leaf et al. [5] previously reported an increasing number of repeats to correlate with an increased risk of AKI in patients that had undergone cardiac surgery. In contrast, in our study the greater number of repeats appeared rather to be protective (see Fig 4). However, the predisposing factors were different in our population. The patients in the study by Leaf et al. had undergone cardiac surgery in cardiopulmonary bypass, omitting emergency surgery, whereas patients in our septic cohort were critically ill septic patients. Additionally, the clinical endpoint of AKI was defined differently. In the study by Leaf the urine output criteria were not included, whereas in our study we chose to exclude patients with mild AKI (KDIGO stage 1) to separate the phenotypes with and without AKI.

The frequency distribution of the dinucleotide repeats showed a bimodal figure with peaks at 23 and 30 alleles (Fig 2), similar to what is presented in previous publications [5,6,15]. This supports the validity of data. To classify the various alleles based on the repeat length, we used the two-class repeat classification, according to the work by Leaf et al [5]. However, three classes have been used [6,12] and hence we made an additional analysis using this three-class repeat classification. This augmented the possibility to detect the association between the very long (>34 repeats) and AKI, which could have possibly driven the results of Leaf et al. No significant associations were found in this additional analysis.

The *HMOX1* promoter repeat polymorphism has been previously associated with critical illness, as has the elevated plasma concentration of HO-1 enzyme [6,12]. The inverse association between a *HMOX1* promoter GTn repeat length polymorphism and the concentration of its gene product, HO-1, has been established repeatedly: the longer the dinucleotide repeat the less HO-1 produced [6,7,16]. Purine-pyrimidine alternating sequences like this have a structure of Z-potential DNA sequence, which is known to negatively affect transcriptional activity [16,17]. The effect of the GTn repeat on gene inducibility by oxidant stress was reported in a transient-transfection assay, concluding that the short repeat allele was more inducible and the basal expression level was higher than in the long repeat allele [16]. We found an association between low HO-1 concentration and very long (>34) repeats. This would concur that only these very long alleles are transcriptionally less active.

HO-1 is an enzyme assisting cells in survival in relation to stress. In an energy-dependent reaction catalyzing heme degradation, HO-1 functions to produce biliverdin along with free iron (Fe) and carbon monoxide (CO) according to the formula: Heme b^2+^ + O_2_ +NADPH + H^+^ → Biliverdin + Fe^2+^ + CO + NADP^+^ + H_2_O. HO-1 plasma concentrations have been shown to associate with cytoprotection in kidneys, but also in other organs [18]. This effect is highly important in defending against exaggerated inflammatory reaction [19–22], but can be inappropriate when found to assist the survival of malignant cell lines [23]. The regulation of HO-1 expression differs between cell and tissue types [16].

In addition to cytoprotection, HO-1 has been found to have a contradicting role as a possible indicator of disease severity, associating with worse outcome in the critically ill [6] and after out-of-hospital cardiac arrest (OHCA) [24]. It has been suggested that there may be an optimal therapeutic range for HO-1 expression in critically ill patients [6,25,26], which could explain the detrimental effects demonstrated with elevated HO-1 levels in this patient group. Moreover, increased HO–1 concentrations are presented in association with AKI [27]. In agreement, we found an indication that lower plasma HO-1 level is associated with a decreased risk of AKI. Despite this, the role of the enzyme induction is commonly seen as protective from AKI [28,29]. Nath et al. [30] published the very first evidence of kidney protection by HO-1 induction already two decades ago, reporting their experiment in a murine model. The function of HO-1 in the presence of critical illness in humans appears to be less simple than that induced experimentally by models mimicking rhabdomyolysis or ischemia-reperfusion in another species. Moreover, in mice the regulation of *HMOX1* expression is different from that of humans [26,31]. In the critically ill the increase in HO-1 plasma concentration is suspected to represent an injury-repairing response against acute illness and thus indicate the magnitude of the damage [6]. In summary, the role of HO-1 enzyme induction in relation to stress remains nebulous [23].

Our study has many strengths. First, the patients have been prospectively and systematically characterized and diagnosed. We defined AKI according to the KDIGO criteria, but the KDIGO-stage-1 patients were omitted from the analysis. We considered that the phenotype of these patients with mild AKI would differ from the more severe AKI, and hence including them could result in biased results. Second, in our sample, both the cases and controls came from the same study cohort and represent critically ill patients with sepsis. Third, we confirmed the reliability of the genotype calling by additional sequencing of 15 homozygote samples. Finally, we included both the two-class and the three-class classifications of allele length in our analyses to achieve a comprehensive view, as both approaches have been utilized in previous studies [5,11,12].

Some limitations of our study need to be considered. First, although the power calculations suggest that we had over 80% power to detect a true association, it might be considered whether the odds ratios in the original work might be inflated and thus, our true power was less than 80%.

Second, the median concentrations of HO-1 were low in comparison to previous studies in septic patients. However, HO-1 levels differed significantly between AKI and no-AKI patients, as well as according to genotype in three-class classification, despite overall modest concentrations. In addition, 52 (8.0%) genotyped patients lacked a plasma sample for enzyme concentration measurement. Finally, we excluded patients with chronic kidney disease (CKD) from the analyses, and thus, our findings are not generalizable to patients with underlying CKD.

We present that in septic, critically ill patients, a small number of dinucleotide repeats in the promoter sequence of *HMOX1* are associated to the development of AKI. Recently, in a phenotype of cardiac-surgery-associated AKI, the inverse association was reported. This finding suggests that the distinction between separate phenotypes within the clinical syndrome of AKI is essential when seeking pathophysiological insight into AKI.

## Supporting information

S1 Appendix Exclusion criteria. (DOCX)

S2 Appendix HO-1 plasma concentration analysis. (DOCX)

S3 Appendix Binary logistic regression for clinical variables and genotypes. (DOCX)

S4 Appendix Disease severity according to genotype. (DOCX)

S5 Appendix HO-1 plasma concentration results. (DOCX)

S6 Appendix Summary level data. (DOCX)
