# Supplementary material for: Heme oxygenase-1 repeat polymorphism in septic acute kidney injury
Source: PLoS One. 2019 May 23;14(5):e0217291. doi: 10.1371/journal.pone.0217291 (PMC6532969; doi:10.1371/journal.pone.0217291)
Supplement: S1 Appendix — (DOCX) [file pone.0217291.s001.docx]

S1 Appendix: Exclusion criteria

Patients excluded from the study were 1) with end-stage renal disease or maintenance dialysis, 2) re-admitted and who previously received renal replacement therapy (RRT), 3) with insufficient language skills or with no permanent residency in Finland, 4) transferred between study ICUs (if already in the study for 5 days), 5) admitted to intermediate care, and 6) organ donors.
