# Supplementary material for: Heme oxygenase-1 repeat polymorphism in septic acute kidney injury
Source: PLoS One. 2019 May 23;14(5):e0217291. doi: 10.1371/journal.pone.0217291 (PMC6532969; doi:10.1371/journal.pone.0217291)
Supplement: S2 Appendix — (DOCX) [file pone.0217291.s002.docx]

S2 Appendix: HO-1 plasma concentration analysis

Heme oxygenase-1 (HO-1) enzyme plasma concentrations were measured using enzyme-linked immunosorbent assay kit (Enzo Life Sciences, Farmingdale, New York), according to the instructions given by the manufacturer. The intra- and inter-assay coefficient of variation (CV%) of the method were determined in a previous study by Siren et al.[1] They were 5.4% for intra-assay and 7.2% for inter-assay CV. Saukkonen et al.[2] have deciphered the reference range for HO–1 plasma concentration (0.66-2.39ng/mL) in 58 healthy subjects. In our data, the inter-assay CV was 19.4% for values less than 1.0ng/mL, and 9.7% for values higher than 1.0ng/mL.

601 samples were included in the HO-1 plasma concentration analysis. In 19 of these samples there were hemolysis present; however, the concentrations are not known to be affected by *in vitro* hemolysis.

1. Siren J, Vaahersalo J, Skrifvars M, Pettilä V, Tiainen M, Tikkanen I, et al. Plasma Heme Oxygenase-1 in Patients Resuscitated from Out-Of-Hospital Cardiac Arrest. Shock. 2016 Nov;45(3):320–5.

2. Saukkonen K, Lakkisto P, Kaunisto MA, Varpula M, Voipio-Pulkki LM, Varpula T, et al. Heme oxygenase 1 polymorphisms and plasma concentrations in critically ill patients. Shock. 2010;34(6):558–64.
