# Supplementary material for: Heme oxygenase-1 repeat polymorphism in septic acute kidney injury
Source: PLoS One. 2019 May 23;14(5):e0217291. doi: 10.1371/journal.pone.0217291 (PMC6532969; doi:10.1371/journal.pone.0217291)
Supplement: S3 Appendix — (DOCX) [file pone.0217291.s003.docx]

S3 Appendix: Binary logistic regression for clinical variables and genotypes

Binary logistic regression for clinical variables

| Logistic regression analysis, AKI vs no AKI | |  |
| --- | --- | --- |
|  | |  |
|  | OR (95% CI) | p-value |
|  |  |  |
| Age* | 1.015 (1.003–1.027) | 0.015 |
| Gender (male) | 0.869 (0.612–1.233) | 0.431 |
| Diabetes | 1.449 (0.959–2.189) | 0.079 |
| Hypertension | 0.945 (0.645–1.383) | 0.796 |
| BMI* | 1.042 (1.015–1.070) | 0.002 |
| COPD | 0.371 (0.200–0.687) | 0.002 |
| Minimum platelet count* | 0.999 (0.997–1.000) | 0.031 |
| SAPS score without age and renal scores* | 1.034 (1.020–1.049) | <0.001 |
| Pseudo R2 (Nagelkerke) |  | 0.126 |

*Odds ratio (OR) per one unit change for continuous variables.

Abbreviations: AKI, acute kidney injury; BMI, body mass index; COPD, congestive obstructive pulmonary disease; SAPS, simplified acute physiology score; HMOX1, heme oxygenase-1 gene; SL, short–long genotype; SS, short–short genotype, LL, long–long genotype; OR, odds ratio, CI, confidence interval.

Binary logistic regression for clinical variables and genotype (1)

| Logistic regression analysis, AKI vs no AKI; genotype SS, SL, LL | |  |
| --- | --- | --- |
|  | OR (95% CI) | p–value |
|  |  |  |
| Age* | 1.014 (1.003–1.026) | 0.017 |
| Gender (male) | 0.865 (0.608–1.229) | 0.417 |
| Diabetes | 1.462 (0.966–2.214) | 0.073 |
| Hypertension | 0.924 (0.630–1.356) | 0.688 |
| BMI* | 1.042 (1.014–1.070) | 0.003 |
| COPD | 0.364 (0.195–0.679) | 0.001 |
| Minimum platelet count* | 0.999 (0.997–1.000) | 0.028 |
| SAPS score without age and renal scores* | 1.035 (1.020–1.050) | <0.001 |
| Genotype SS vs LL | 1.810 (1.005–3.257) | 0.048 |
| Genotype SL vs LL | 1.174 (0.821–1.679) | 0.374 |
| Pseudo R2 (Nagelkerke) |  | 0.134 |

*Odds ratio (OR) per one unit change for continuous variables.

Abbreviations: AKI, acute kidney injury; BMI, body mass index; COPD, congestive obstructive pulmonary disease; SAPS, simplified acute physiology score; HMOX1, heme oxygenase-1 gene; SL, short–long genotype; SS, short–short genotype, LL, long–long genotype; OR, odds ratio, CI, confidence interval.

Binary logistic regression for clinical variables and genotype (2)

| Logistic regression analysis, AKI vs no AKI; genotype SS, SM, SL_2_, MM, ML_2_ (no AKI patients in L_2_L_2_ genotype) | |  |
| --- | --- | --- |
|  | OR (95% CI) | p–value |
|  |  |  |
| Age* | 1.014 (1.002–1.026) | 0.021 |
| Gender (male) | 0.854 (0.600–1.216) | 0.381 |
| Diabetes | 1.452 (0.958–2.199) | 0.079 |
| Hypertension | 0.934 (0.636–1.371) | 0.726 |
| BMI* | 1.043 (1.015–1.071) | 0.002 |
| COPD | 0.369 (0.197–0.689) | 0.002 |
| Minimum platelet count* | 0.999 (0.997–1.000) | 0.028 |
| SAPS score without age and renal scores* | 1.035 (1.020–1.050) | <0.001 |
| Genotype SM vs SS** | 0.672 (0.375–1.202) | 0.180 |
| Genotype MM vs SS** | 0.552 (0.304–1.002) | 0.051 |
| Genotype SL_2_ vs SS** | 0.415 (0.145–1.189) | 0.101 |
| Genotype ML_2_ vs SS** | 0.590 (0.242–1.436) | 0.245 |
| Pseudo R2 (Nagelkerke) |  | 0.138 |

*Odds ratio (OR) per one unit change for continuous variables.

** Reference set to SS genotype, because no AKI cases in L_2_L_2_ genotype.

Abbreviations: AKI, acute kidney injury; BMI, body mass index; COPD, congestive obstructive pulmonary disease; SAPS, simplified acute physiology score; HMOX1, heme oxygenase-1 gene; SL, short–long genotype; SS, short–short genotype, LL, long–long genotype; OR, odds ratio, CI, confidence interval.
