# Supplementary material for: Heme oxygenase-1 repeat polymorphism in septic acute kidney injury
Source: PLoS One. 2019 May 23;14(5):e0217291. doi: 10.1371/journal.pone.0217291 (PMC6532969; doi:10.1371/journal.pone.0217291)
Supplement: S4 Appendix — (DOCX) [file pone.0217291.s004.docx]

S4 Appendix: Disease severity according to genotype

| Genotype, two alleles | Median SAPSII score without renal and age points | IQR |
| --- | --- | --- |
| SS | 26.0 | 20.0–31.0 |
| SL | 24.0 | 17.0–32.0 |
| LL | 25.0 | 19.0–35.0 |

| Genotype, three alleles | Median SAPSII score without renal and age points | IQR |
| --- | --- | --- |
| SS | 26.0 | 20.0–31.0 |
| SM | 24.0 | 17.0–31.5 |
| MM | 25.0 | 18.5–35.0 |
| SL2 | 24.0 | 20.0–32.0 |
| ML2 | 24.5 | 19.0–30.0 |

Abbreviations: SAPS, simplified acute physiology score; IQR, interquartile range.
