# Supplementary material for: Heme oxygenase-1 repeat polymorphism in septic acute kidney injury
Source: PLoS One. 2019 May 23;14(5):e0217291. doi: 10.1371/journal.pone.0217291 (PMC6532969; doi:10.1371/journal.pone.0217291)
Supplement: S5 Appendix — (DOCX) [file pone.0217291.s005.docx]

S5 Appendix: HO-1 plasma concentration results

Of the 601 patients with HO-1 plasma concentration measured, 277 (46%) had AKI and 324 (54%) did not have AKI. Median HO-1 concentration for patients with AKI was 2.3ng/mL (IQR 1.1–4.2ng/mL), and for patients without AKI 1.7ng/mL (IQR 1.0–2.6ng/mL); the concentration of patients with AKI was significantly higher (*p*=0.001). Of the 601 patients 62 (10%) had genotype SS, 295 (49%) had genotype SL, and 244 (41%) had genotype LL. Median HO-1 concentration for patients with SS genotype was 1.8ng/mL (IQR 0.8–2.5ng/mL), for patients with SL genotype 1.9nanog/mL (IQR 1.1–3.3ng/mL), and for patients with LL genotype 1.9nanog/mL (IQR 1.0–3.4ng/mL); the concentrations did not significantly differ according to patient genotype (*p*=0.35).
