# Supplementary material for: Heme oxygenase-1 repeat polymorphism in septic acute kidney injury
Source: PLoS One. 2019 May 23;14(5):e0217291. doi: 10.1371/journal.pone.0217291 (PMC6532969; doi:10.1371/journal.pone.0217291)
Supplement: S6 Appendix — (DOCX) [file pone.0217291.s006.docx]

S6 Appendix: Summary level data

| Genotype frequencies (two-class classification) | |  | No AKI | | | AKI KDIGO Stage 2 | | | AKI KDIGO Stage 3 | | |
| --- | --- | --- | --- | --- | --- | --- | --- | --- | --- | --- | --- |
| Variant | minor allele (b) | major allele (B) | bb | Bb | BB | bb | Bb | BB | bb | Bb | BB |
| HMOX1 GTn | S | L | 8.5% | 48.2% | 43.3% | 12.9% | 44.6% | 42.6% | 13.6% | 51.8% | 34.7% |

| Genotype frequencies  (three-class classification) | | | | | |  | | | | | | |  | | | | | |
| --- | --- | --- | --- | --- | --- | --- | --- | --- | --- | --- | --- | --- | --- | --- | --- | --- | --- | --- |
| No AKI | | | | | | AKI KDIGO Stage 2 | | | | | | | AKI KDIGO Stage 3 | | | | | |
| SS | SM | MM | SL2 | ML2 | L2L2 | | SS | SM | MM | SL2 | ML2 | L2L2 | SS | SM | MM | SL2 | ML2 | LL22 |
| 8.5 % | 43.3 % | 37.4 % | 4.8 % | 5.7 % | 0.3 % | | 12.9 % | 41.6 % | 38.6 % | 3.0 % | 4.0 % | 0.0 % | 13.6 % | 48.7 % | 28.6 % | 3.0 % | 6.0 % | 0.0 % |
